# Dendrimer and dendrimer gel-derived drug delivery systems: Breaking bottlenecks of topical administration of glaucoma medications

**DOI:** 10.1002/mba2.30

**Published:** 2023-02-20

**Authors:** Juan Wang, Boxuan Li, Uday B. Kompella, Hu Yang

**Affiliations:** 1 College of Biomedical Engineering, Sichuan University, Chengdu, Sichuan, China; 2 Tianjin Key Laboratory of Biomedical Materials, Key Laboratory of Biomaterials and Nanotechnology for Cancer Immunotherapy, Institute of Biomedical Engineering, Chinese Academy of Medical Sciences & Peking Union Medical College, Tianjin, China; 3 Department of Pharmaceutical Sciences, University of Colorado Anschutz Medical Campus, Aurora, Colorado, USA; 4 Linda and Bipin Doshi Department of Chemical and Biochemical Engineering, Missouri University of Science and Technology, Rolla, Missouri, USA

**Keywords:** dendrimer, dendrimer hydrogel, dendrimer micro/nanogel, drug delivery, glaucoma

## Abstract

Due to high structural flexibility, multidrug carrying capability, and tunable size, dendrimers have been used as suitable carriers for ophthalmic drug delivery. Drug molecules can be either encapsulated or chemically coupled to dendrimers. The nanoscopic size, spheroidal shape, and cationic surface of polyamidoamine (PAMAM) dendrimers promote their interaction with the cornea and result in prolonged precorneal retention. Dendrimers could be further cross-linked to produce three-dimensional hydrogel networks or dendrimer hydrogels (DH). The properties of the DH can be readily adjusted to maintain both fluidity and adhesiveness, making them suitable for developing topical ocular drug formulations. Micro-/nano-sized DHs, that is, dendrimer micro-/nano-gels, have unique properties such as ease of administration, large specific surface area for adhesion, and drug targeting functionalities, making them attractive for ophthalmic drug delivery. This perspective reports advances in PAMAM dendrimer based drug delivery systems including drug conjugates and micro- and nano-gels to enhance and sustain the delivery of multiple anti-glaucoma drugs, Dendrimer and dendrimer gel-derived drug delivery systems hold great potential as multifunctional topical drug delivery systems for the eye.

## 1 | INTRODUCTION

**FIGURE 1:**
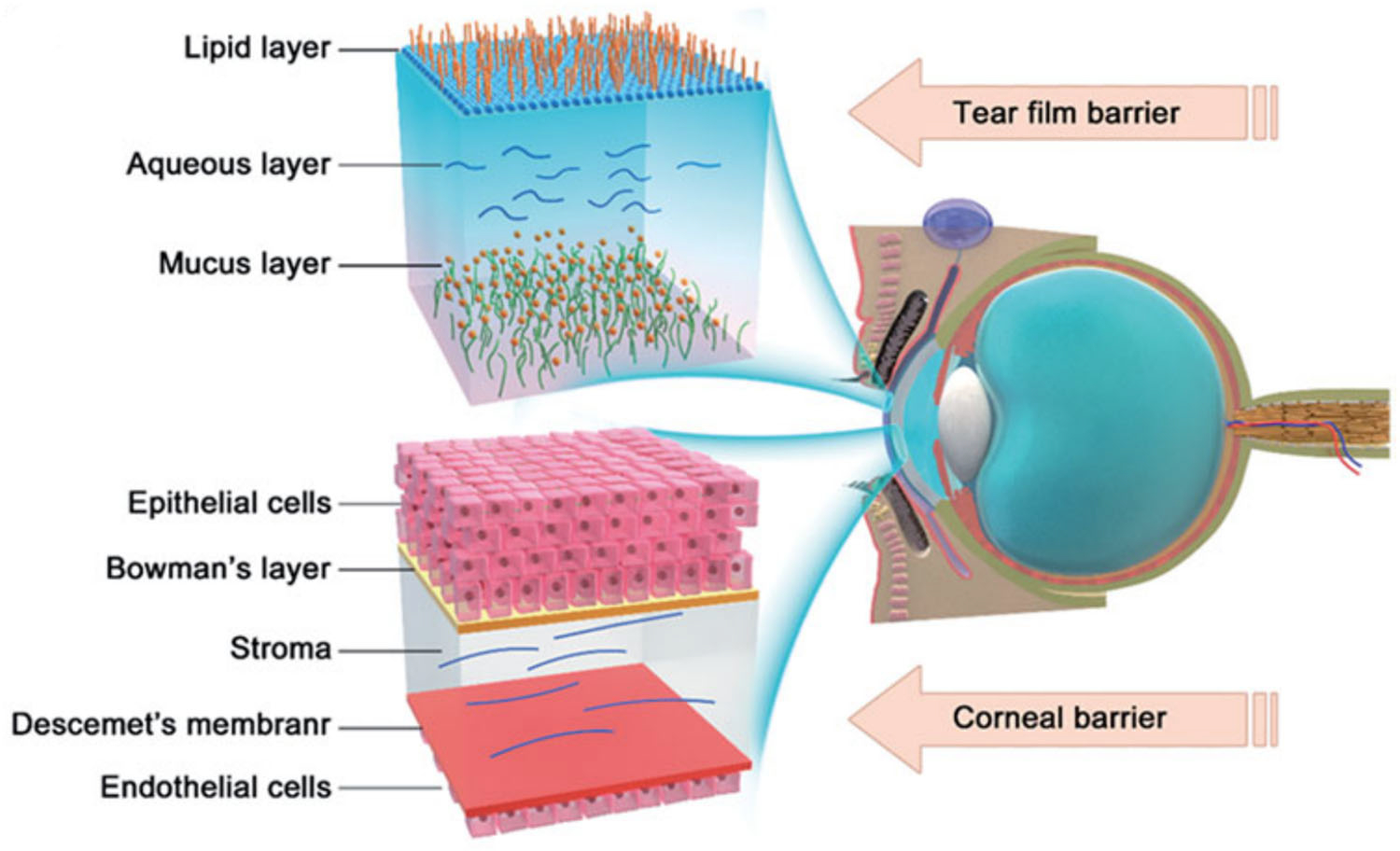
Two major barriers to topical ocular drug delivery. Reprinted with permission from Li et al.^7^

Glaucoma, a group of slowly progressive eye diseases/disorders characterized by elevated intraocular pressure (IOP), has become the leading cause of irreversible blindness worldwide.^1,2^ Clinically used medications for the treatment of glaucoma can be grouped into two major types based on their modes of action. α-adrenergic agonists, *β*-blockers, and carbonic anhydrase inhibitors reduce the aqueous humor production, while prostaglandins, rho kinase inhibitors, nitric oxides, and miotic or cholinergic agents help fluid drain from the eye.^2,3^ Due to the complexity of glaucoma etiology, a single medication is often insufficient in reducing IOP. Clinically, two or more drugs with distinct modes of action are often needed to achieve and maintain IOP-lowering effects for the long term.^4^ A variety of combination regimens have been widely used in the treatment of glaucoma.^5^ Among all the current treatments to reduce IOP for glaucoma therapy, eye drop instillation is the most broadly used mode of administration.^6^ Nevertheless, due to the tear flow and corneal permeability barriers (Figure 1), the topically administered glaucoma medications usually encounter serious precorneal drug loss and low corneal penetration.^7^ In addition, most drugs absorbed through the conjunctiva enter the systemic circulation, while the target tissue of the IOP-lowering drug is mostly in the anterior chamber, which further reduces the target bioavailability of the drug.^8,9^

**FIGURE 2:**
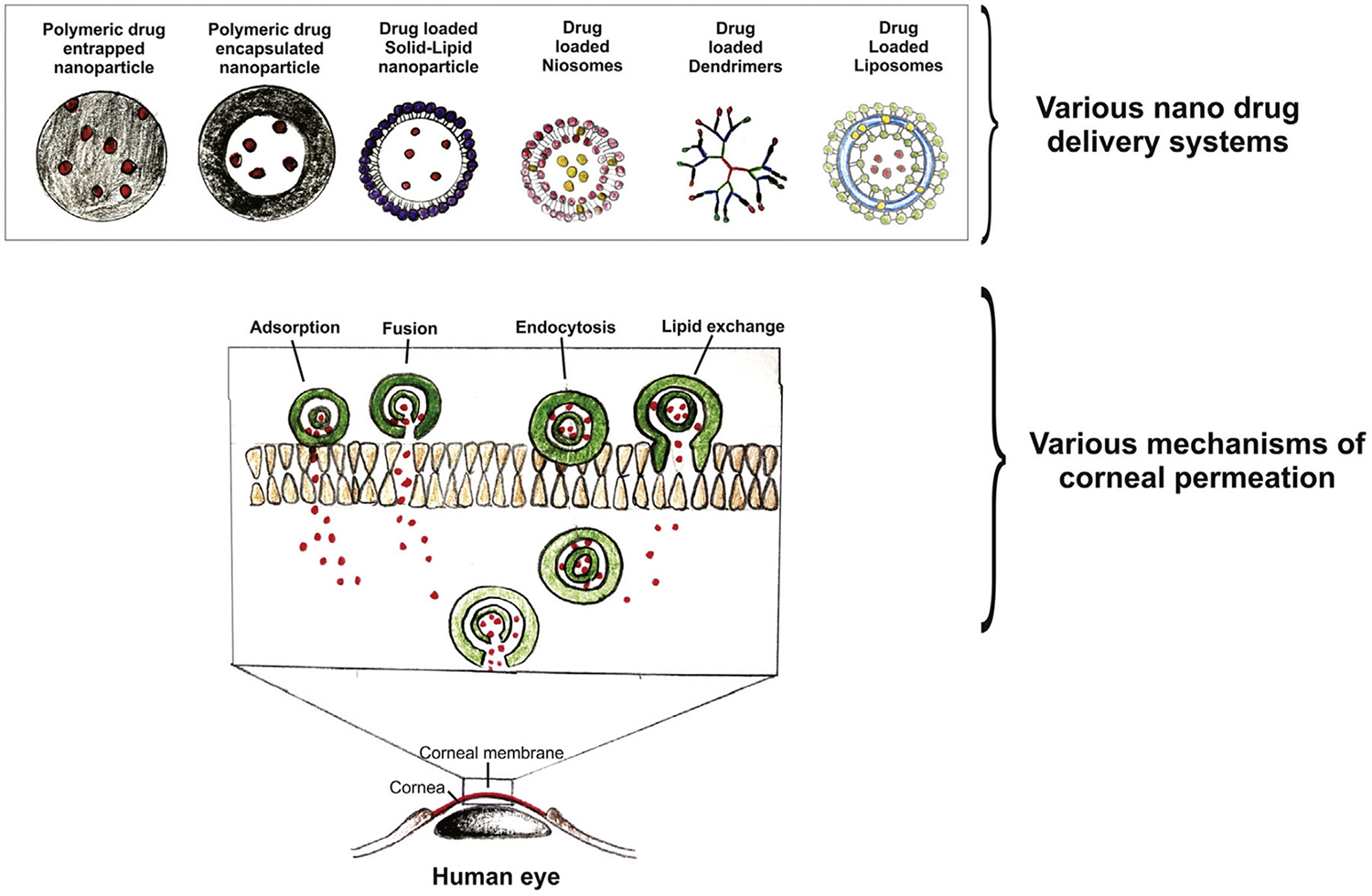
Various nano-drug delivery systems for corneal permeation. Reprinted with permission from Yadav et al.^2^ Copyright 2019 of Elsevier.

To overcome low drug bioavailability and realize multiple drug delivery following topical administration, drug delivery systems have been extensively studied because they hold great potential to reduce precornea loss, improve drug permeation across the cornea, and enable multidrug loading, targeted delivery, and sustained drug release. So far, commonly assessed delivery carriers mainly include (1) cationic carriers; (2) viscous media, such as gels; and (3) nanoparticles. The cornea is negatively charged at physiological pH. When used as ophthalmic drug delivery carriers, cationic polymers and cationic micro- and nano-particles have an excellent corneal affinity, extending corneal retention time.^10^ Some in situ hydrogels have excellent corneal adhesion/retention and have been used as glaucoma drug carriers.^11–13^ However, the conventional gel systems may cause vision blurring due to uneven cross-linking on the ocular surface. Recently, Kim et al. reported a low osmotic in situ gel solution. It forms a highly uniform and transparent gel film on the ocular surface. It can avoid blurred vision and prolong the retention time for achieving long-term drug effects.^14^ Although hydrogels can improve corneal adhesion and achieve sustained drug release, there are still shortcomings in improving the corneal permeability of drugs. Nanoparticles are conducive to improving corneal permeability of poorly permeable drugs because of their small size and flexible surface functionalization that may enhance cell entry.^15–19^ So far, a variety of organic and inorganic nanoparticles were assessed as delivery vehicles for glaucoma treatment (Figure 2).^2,20^ Sun et al. used mesoporous silica nanoparticles to deliver nitric oxide donors to the trabecular meshwork and Schlemm’s canal.^21^ The delivery system maintained a long-term IOP reduction effect. By combining the advantages of hydrogel and nanoparticles, nanogels may have good corneal adhesion, corneal permeability, and sustained drug release ability, without blurring vision. Therefore, nanogels, especially positively surface-charged nanogels, are potential candidates as topical drug delivery systems for glaucoma therapy.

Although current drug delivery systems have made progress in improving drug bioavailability, a delivery vehicle that can combine the advantages of a cationic carrier, gel, and nanoparticle is still rarely developed and reported. To comprehensively improve the bioavailability of drugs and achieve long-term IOP reduction effect, developing a multifunctional drug delivery system to accommodate the variety of mechanisms of action and target tissues is the key to overcoming the bottlenecks in topical delivery of glaucoma medications. We focus on the advantages of dendrimer and dendrimer gel-derived drug delivery systems for topical administration. We hope that this perspective will provide deep insights into the design of novel dendrimer-derived topical drug delivery system (TDDS) for efficient glaucoma therapy.

## 2 | DENDRIMER-BASED OCULAR DRUG DELIVERY SYSTEMS

### 2.1 | Dendrimer is a suitable candidate for ophthalmic drug delivery

A dendrimer is a well-defined branched macromolecule with a unique three-dimensional nanostructure consisting of tree-like arms or branches built around a small molecule or a linear polymer core.^22^ The dendrimer’s size, surface charge, peripheral functional groups, and solubility can be tuned. Dendrimers have shown promising mucosal adhesion properties, facilitating long-term precorneal retention. Bravo-Oscuna et al. reported that polyamidoamine (PAMAM) dendrimers presented strong interfacial interactions with transmembrane ocular mucins at physiological and pathological tear pH using a variety of surface chemical groups.^23^ A cationic carbosilane dendrimer was synthesized and found to have strong interaction with ocular mucins, making this dendrimer a useful delivery carrier to increase drug retention time on the ocular surface.^24^ Amine-terminated PAMAM dendrimers, one of the most used and commercial dendrimers, possess a cationic periphery and hold the potential to increase corneal permeability when applied in an aqueous solution.^25^,^26^ Dendrimers offer a high degree of flexibility in drug carrying, thereby enabling multidrug loading for efficient glaucoma therapy. Drugs can be either physically encapsulated into the dendrimer inner core, complexed with dendrimer peripheral by electrostatic interactions, or chemically conjugated to the dendrimer surface. The multivalent properties of dendrimers make them ideal for creating hierarchical structures, such as hydrogels and micro- or nano-gels, thus enriching their functionality as ocular drug delivery carriers. In addition, recent studies have shown that glaucoma is associated with inflammation in addition to elevated IOP.^27^ Amine-terminated PAMAM dendrimers exhibit anti-inflammatory capacity, therefore, when used as drug delivery vehicles, they may inhibit inflammatory mediators.^27,28^

**FIGURE 3:**
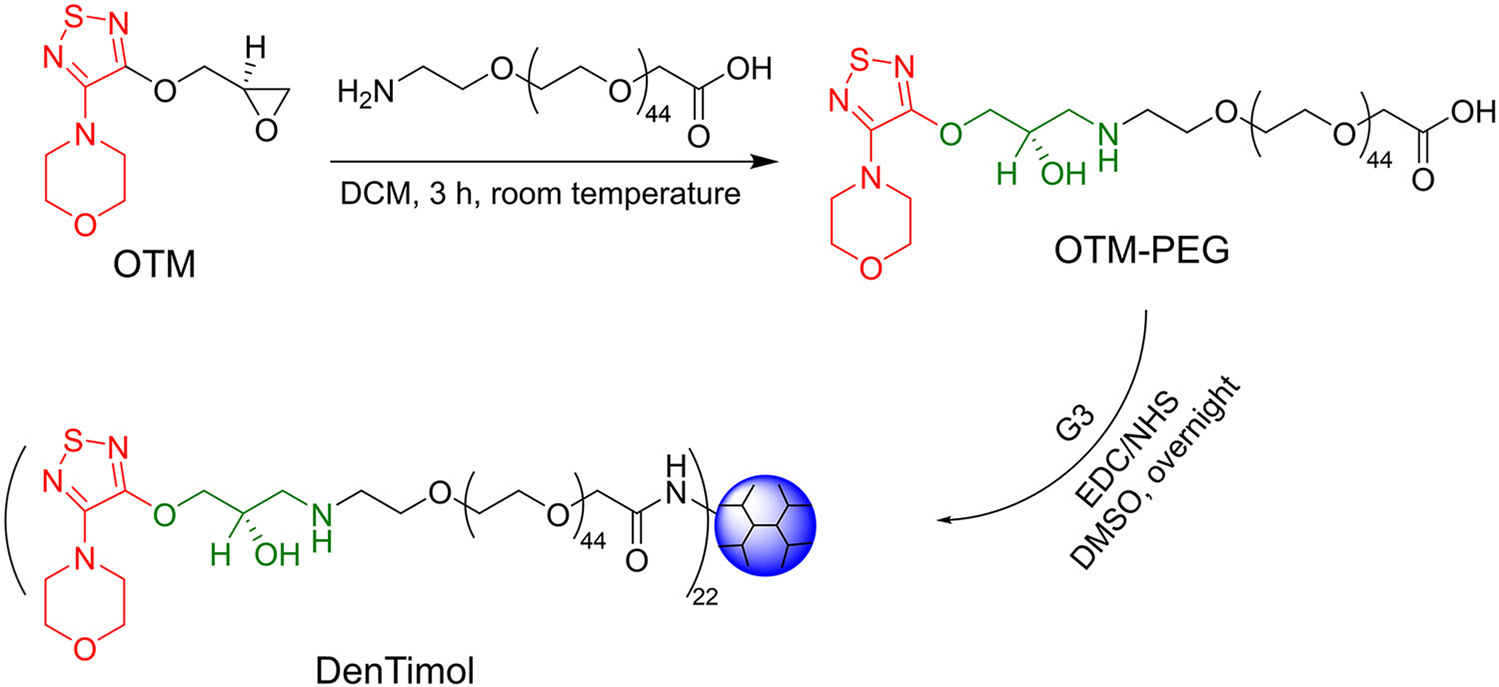
Synthesis of DenTimol. Reprinted with permission from Li et al.^18^ Copyright 2018 of American Chemical Society.

Mishara et al. used generation 5 of poly(propylene imine) dendrimer (PPI G5) to physically encapsulate acetazolamide to treat glaucoma. Due to its nanoscopic size, surface charge, and spherical shape, PPI showed prolonged ocular residence time, ultimately facilitating sustained IOP lowering effect.^29^ Brimonidine tartrate (BT) was physically encapsulated with PAMAM G3 and then subjected to electrospinning to form a nanofiber.^30^ This nanofiber dissolved fast and enhanced BT adsorption when topically administrated to the eye. The dendrimer-drug conjugate, DenTimol, was synthesized, and the therapeutic efficacy of DenTimol for glaucoma treatment was studied in our previous work (Figure 3).^31^ DenTimol was composed of a precursor of the commercial antiglaucoma drug, (S)-4-[4-(oxiranylmethoxy)-1,2,5-thiadiazol-3-yl]morpholine, conjugated to PAMAM G3 dendrimer surface through a polyethylene glycol (PEG) spacer. Possibly due to the suitable mucoadhesive property of the dendrimer, DenTimol is efficient at crossing the cornea, and about 8% of the dendrimer-drug permeated through the cornea in 4 h. DenTimol demonstrated a stronger IOP-lowering effect than timolol maleate (TM) in normotensive adult Brown Norway male rats.

### 2.2 | Dendrimer hydrogel ocular drug delivery systems enhance antiglaucoma drug delivery

Hydrogels are high-water content materials commonly prepared from chemically or physically cross-linked polymers.^32^ Hydrogels are widely applied in the field of biomedicine, and they are able to provide sustained, local delivery of a variety of therapeutic agents.^33^ Dendrimer could be used as an ideal building block for producing three-dimensional cross-linked networks due to its multifunctionality and well-defined nanostructure.^34,35^ Various approaches, including physical crosslinking strategies and adopting chemical cross-linkers, were utilized for the fabrication of dendrimer hydrogels.^36^ Buwalda et al. fabricated PAMAM dendrimer hydrogel by using multiarmed PEG star polymers bearing *N*-succinimidyl ester end groups as a cross-linker.^37^ The gelation process was fast, and the swelling, degradation, and mechanical properties of the dendrimer hydrogel could be readily modulated. Compared with the dendrimer itself, dendrimer hydrogels enrich the ways of using dendrimers for drug delivery. Drugs can be carried by dendritic building blocks or in situ embedded in the hydrogel network.^38^ In addition, the precorneal retention time may be further prolonged due to the adhesion property of hydrogels. Nguyen et al. prepared a thermogel by using PAMAM, gelatin, and poly(*N*-isopropylacrylamide).^27^ The thermogel showed excellent drug encapsulation properties and sustained drug release, inhibited inflammation, and resulted in a prolonged IOP lowering effect as long as 84 days.

**FIGURE 4:**
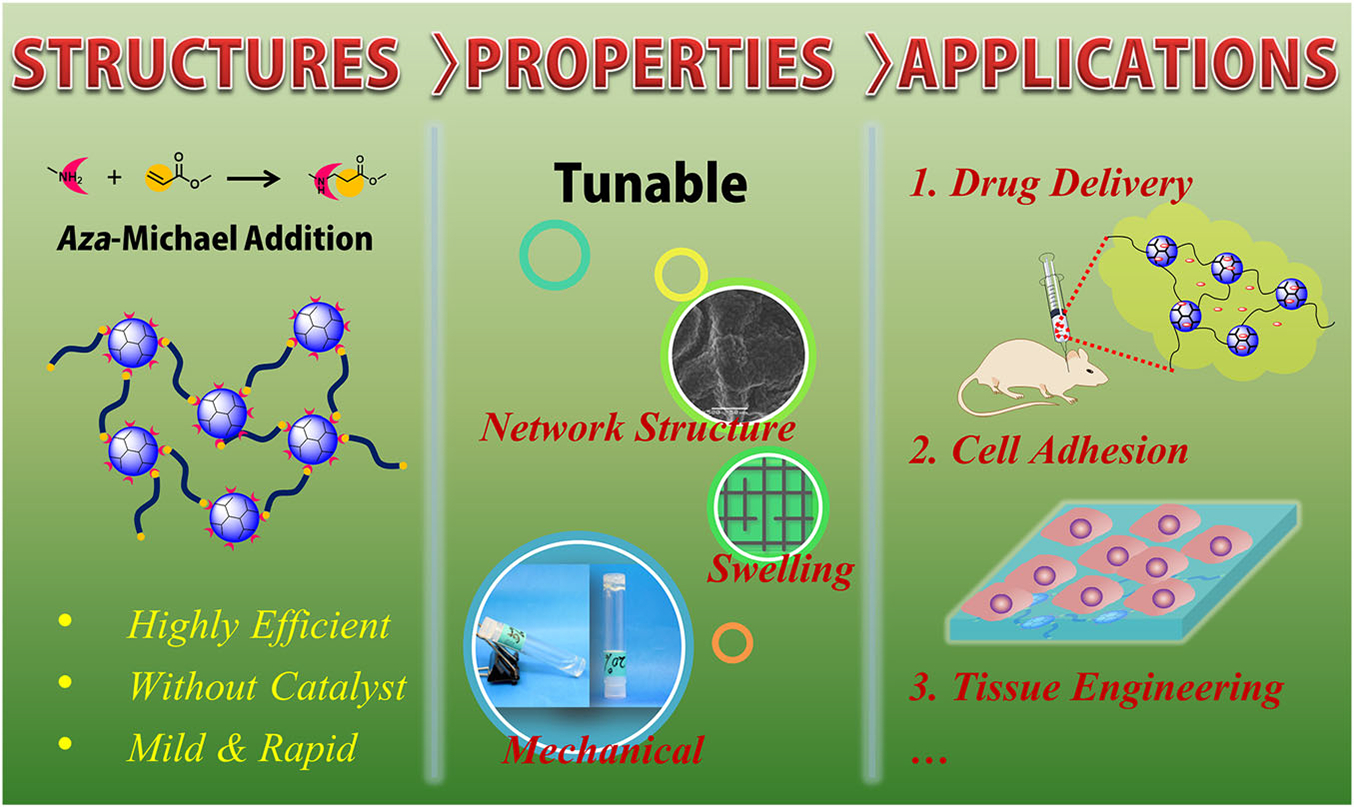
A dendrimer hydrogel (DH) platform prepared through the aza-Michael addition cross-linking strategy. Reprinted with permission from Wang et al.^39^ Copyright 2017 of American Chemical Society.

**FIGURE 5:**
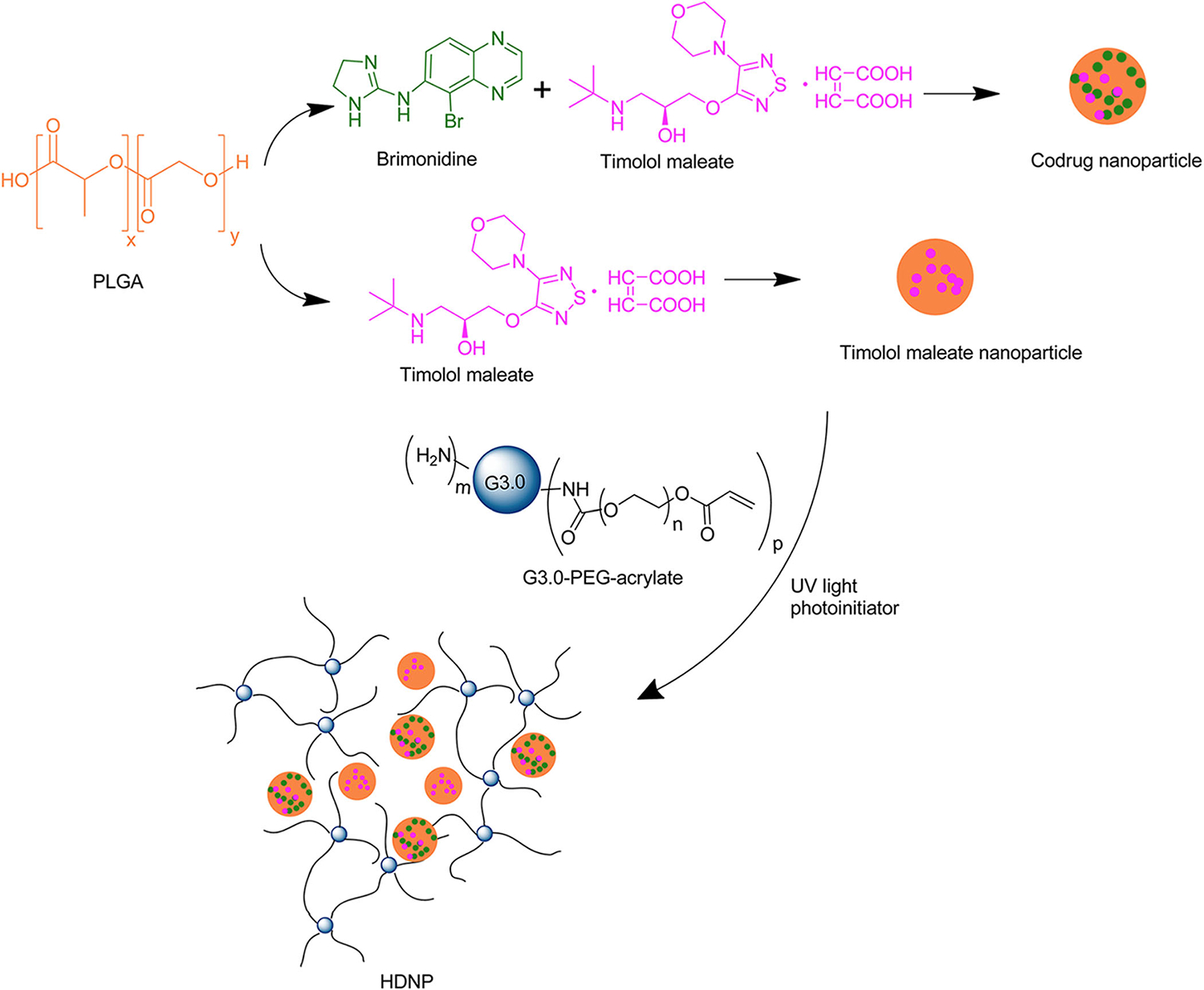
The hybrid dendrimer hydrogel/nanoparticle platform. Reprinted with permission from Yang et al.^41^ Copyright 2012 of American Chemical Society.

Based on the PAMAM dendrimer, we developed dendrimer hydrogel (DH) through the aza-Michael addition cross-linking strategy (Figure 4).^39^ In this DH, PAMAM dendrimer is the main component, and polyethylene glycol diacrylate (PEG-DA) was chosen as a biocompatible cross-linker. In the cross-linking reaction, the nucleophilic amines on the PAMAM dendrimer surface react with the unsaturated ester of the terminal acrylate groups in linear PEG-DA to form a three-dimensional structure. This dendritic-linear cross-linking strategy is a green approach since the aza-Michael addition reaction proceeds efficiently in aqueous media at room temperature without using any catalyst or generating any by-product. Hydrogels prepared by photoinitiated crosslinking have been broadly reported by virtue of their easy handling, and rapid gelation and part of them have been explored as biomedical materials.^34,40^ Our group has also synthesized dendrimer hydrogels using a photopolymerization strategy previously. PEGylated PAMAM G3 was first surface-modified to possess acrylate end-groups and then cross-linked by using an eosin Y photoinitiating system.^35^ We synthesized a hybrid PAMAM dendrimer hydrogel/poly(lacticco-glycolic acid) nanoparticle platform for the codelivery of two antiglaucoma drugs, BT and TM, by using the same photopolymerization strategy (Figure 5).^41^ The system significantly reduced the dosing frequency of topical formulations and it might improve long-term patient compliance. Photoinitiated cross-linking inevitably requires photoinitiators. The potential cytotoxicity of photoinitiators may hinder the development of photocurable hydrogel systems in the biomedical field. Xu et al. studied the cytotoxicity and intracellular AKT expression of three photoinitiators, including 2,2-dimethoxy-2-phenylacetophenone, Irgacure 2959, and eosin Y photoinitiating system in HN4 cells.^42^ The three photoinitiators affect intracellular AKT signaling, which in turn causes cytotoxicity in a dose-dependent manner. Therefore, the use and dosage of photoinitiators needs caution. Different from the photoinitiated hydrogels, following the aza-Michael addition, our DH requires no trigger of agent, light, or heat, avoiding the biosafety issues caused by initiators. In addition, the aza-Michael addition DH is an in situ-formed gel and thus could be used as an injectable hydrogel. As a delivery system, this DH has the advantage that it could maintain the liquid nature or fluidity for convenient injection or drop instillation and in situ formed gel after topical administration for sustained delivery. The properties of the PAMAM-based DH can be readily adjusted by the degree of cross-linking through controlling reactant concentration or the amine group density on the surface of PAMAM.

The PAMAM dendrimer-based DH has many advantages of being a delivery system. The DH has better biocompatibility compared to free dendrimers in equivalent molar qualities due to the cross-linking with PEG. Due to the positively charged PAMAM dendrimer and the hydrogel’s adhesiveness, the DH possesses an excellent corneal affinity for ocular delivery. Based on our strategy, a mildly cross-linking PAMAM dendrimer hydrogel (mcDH) was constructed as eye drops by choosing the antiglaucoma drug BT as a model drug.^43^ This mcDH exhibited both fluidity and adhesiveness, making it a suitable system for developing topical ocular drug formulation. A significantly sustained BT release and an enhanced corneal permeability are realized from the mcDH. BT could be burst released from the cross-linked network of the mcDH, and more sustained release from the dendrimer inner core.

### 2.3 | Dendrimer micro-/nano-gels combine the advantages of dendrimer, hydrogel, and nanoparticle

Micro-/nano-gels are suited for hydrogels that need an injection or require smaller sizes.^44–49^ Compared to bulk hydrogels, micro-/nano-gels have many unique properties, such as easy administration, large specific surface area for adhesion, and drug targeting, making them attractive for biomedical applications. Pilocarpine was loaded in a pH-sensitive polyvinylpyrrolidone-poly (acrylic acid) nanogel through electrostatic interactions with high loading efficiency.^50^ Compared with the pilocarpine solution, this pilocarpine-loaded nanogel exhibited sustained release. By virtue of the intrinsic mucoadhesive property of poly(acrylic acid), the nanogel showed good precorneal mucin affinity. A redox-responsive hyaluronic acid-based nanogel was prepared and used to deliver therapeutics to the posterior segment by topical instillation.^51^ The drug release was triggered by the reducing environment. It was the first reported topically applied triggered-release drug delivery system that can target the pigmented layer of the retina after topical administration. Based on our strategy for exploiting DH, we further developed PAMAM-based micro-/nano-gels by combining the aza-Michael addition cross-linking reaction with the inverse micro-/nano-emulsion method.^52,53^ The reacting system of the dendrimer is dissolved in water first and then converted into micro-/nano-droplets by dispersing in a continuous organic phase in the presence of surfactants. The gelation process between dendrimer and PEG-DA occurs in the micro-/nano-droplets. Microgel and nanogel particles with sizes ranging from several micrometers to hundreds of nanometers were successfully prepared by changing preparation parameters.

Dendrimer microgels (μDHs) prepared by us have a size of 3–5 μm with a relatively narrow size distribution.^52^ They show good cell internalization and excellent cytocompatibility and do not cause acute toxicity in vivo. They can realize a high loading of drugs and enter the cells in the form of particles. The μDHs are pH-responsive and degradable, and the drug can be slowly released with zero-order release kinetics. The μDHs could be seen as a new class of microparticles with a hierarchically ordered PAMAM dendrimer in micrometer domains. They show an expanded structural feature for programmable drug delivery and release.

**FIGURE 6:**
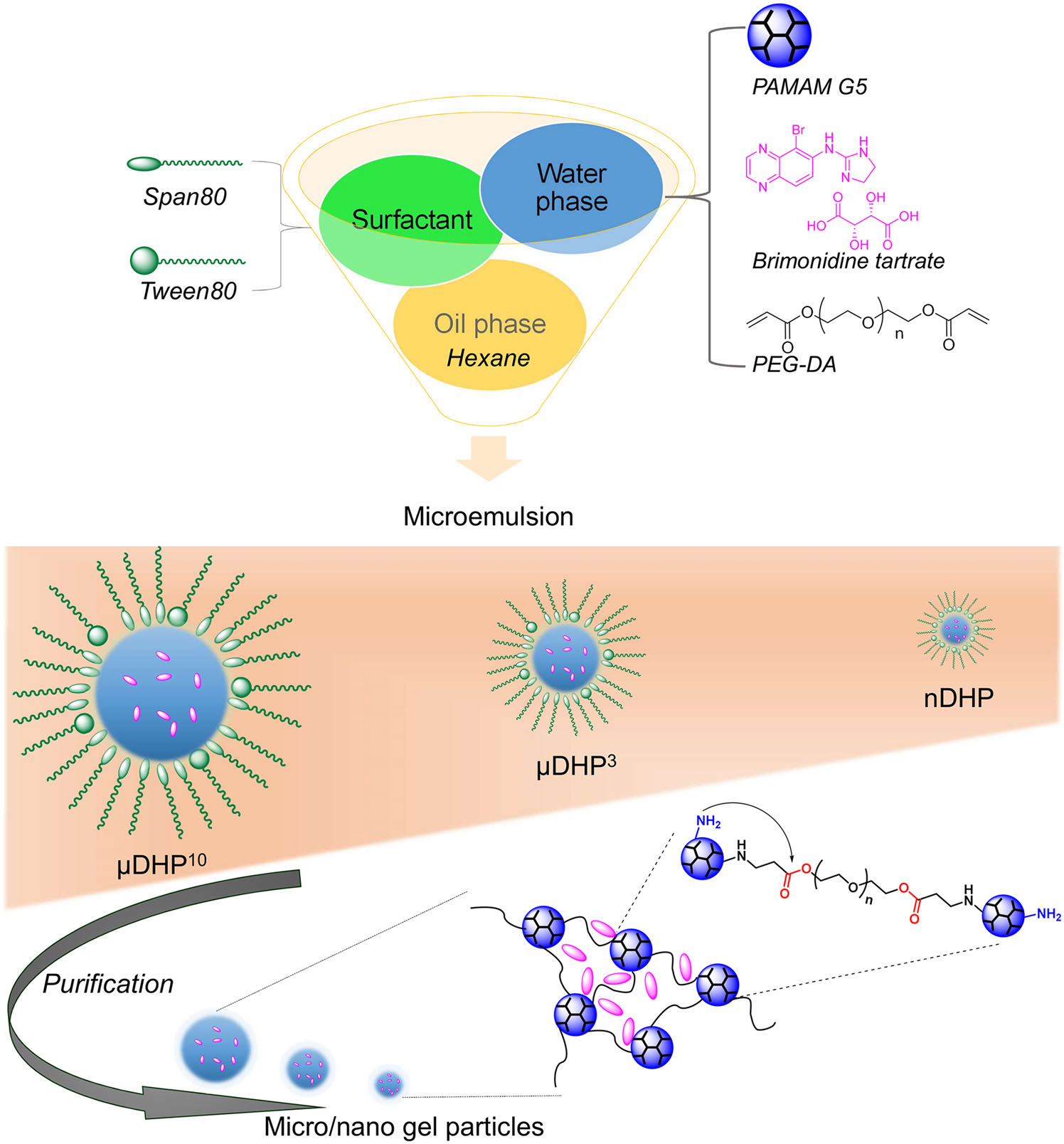
Schematic illustration of the preparation of dendrimer gel particles of various sizes by using the invert emulsion method coupled with aza-Michael addition reaction. Figure modified and reprinted with permission from Wang et al.^53^ Copyright 2021 of Elsevier.

Nanogels with a smaller size could more obviously reflect the unique feathers from both particles and hydrogels. Recently, we further identified parameters of the inverse emulsion method after the investigation for preparing μDHs, and we could successfully obtain a PAMAM-based dendrimer micro/nanogel with a size range from tens of micrometers to nanometers (Figure 6).^52,53^ A PAMAM-based dendrimer hydrogel nanoparticle (nDHP) with a size of about 200 nm was used as a drug carrier for the topical delivery of antiglaucoma drugs. Two first-line antiglaucoma drugs, BT and TM were loaded into dendrimer hydrogel nanoparticles. The nDHP was found to be superior to microgel drug-carrying systems in terms of cytocompatibility, degradability, drug release kinetics, and corneal permeability. With the help of nDHPs, the drug corneal permeability was increased by 17-fold compared to plain drug solution, and zero-order prolonged drug release kinetics were revealed. Pronounced IOP-lowering effects were found by using the nDHP-based formulation in both single-dose tests and 7-day chronic daily dosing tests in both Brown Norway rats and glaucoma mice. Since the dendrimer itself is a nano-sized macromolecule, the dendrimer hydrogel nanoparticles could be seen as a nano-in-nano drug-carrying system. Benefiting from dendrimer, hydrogel, and particles, the developed nano-in-nano dendrimer gel particles could precisely dose and enable sustained and synergistic efficacy of antiglaucoma drugs for improving glaucoma treatment.

## 3 | CONCLUSION AND PERSPECTIVES

The ultimate goal of glaucoma therapy is to achieve long-term IOP reduction with low administration frequency. The development of multifunctional drug delivery systems is the key to enhance and sustain delivery of a combination of glaucoma drugs. The design of drug carriers should reflect the multiple functions of extending corneal retention, enhancing corneal permeability, high loading efficiency, and controllable release of different drugs. At the same time, accurate drug-targeting should be realized. Several drugs are to be delivered to the anterior chamber via the corneal or noncorneal pathways, and then to target anterior chamber tissues according to the mechanism of drug action. Such drug delivery carriers can fundamentally improve the bioavailability and/or persistence of drugs, thereby reducing the frequency of drug dosing and maintaining long-term intraocular pressure reduction.

Dendrimer gel-derived drug delivery systems could be multifunctional TDDS, integrating the advantages of dendrimer, gel, and nanoparticles. By virtue of the nanosize, positive surface charge, and surface functionalization, it is possible to reduce pre-corneal drug loss, prolong the retention on the cornea and enhance the permeation across the cornea. Multiple drugs with distinct modes of action could co-loaded by physical embedding and/or chemical coupling. Drugs could be accurately targeted to their action site followed by a controlled release. Dendrimer has well-defined molecular weight and architecture. Compared with other commonly used nano-drug delivery carriers, such as polymeric nanoparticles and liposomes, the structure of dendrimer-derived drug delivery carriers might have better controllability and stability. In addition, the intrinsic inflammation inhibition capacity of dendrimer helps better glaucoma therapy. Thus, the dendrimer-gel-based TDDS holds great potential to realize a continuous reduction of IOP with a low frequency of administration, thereby improving therapeutic efficacy and patient compliance. The main challenges that restrict the development of dendrimer-derived TTDS may include (1) in vivo long-term safety, (2) in vivo biodistribution and metabolism, and (3) cost of mass production. Despite these challenges, we believe that novel dendrimer and dendrimer gel-derived TDDS could be used for topical ocular drug delivery with potent antiglaucoma efficacy.

## Data Availability

Not applicable.

## References

[1] Tham YC, Li X, Wong TY, Quigley HA, Aung T, Cheng CY. Global prevalence of glaucoma and projections of glaucoma burden through 2040. Ophthalmology. 2014;121(11):2081–2090. 24974815 10.1016/j.ophtha.2014.05.013

[2] Yadav KS, Rajpurohit R, Sharma S. Glaucoma: current treatment and impact of advanced drug delivery systems. Life Sci. 2019;221:362–376. 30797820 10.1016/j.lfs.2019.02.029

[3] Guo XJ, Chen D, Wu HX, Retrospective study of topical medical treatment for glaucoma. Int Eye Sci. 2018;18:1893–1897.

[4] Lam D, Lee J, Jonas J, Glaucoma: today and tomorrow. Asia-Pacific J Ophthalmol. 2016;5(1):2–4. 10.1097/APO.0000000000000184 26886113

[5] Schehlein EM, Novack GD, Robin AL. New classes of glaucoma medications. Curr Opin Ophthalmol. 2017;28(2):161–168. 27828896 10.1097/ICU.0000000000000346

[6] Patel A Ocular drug delivery systems: an overview. World J Pharmacol. 2013;2(2):47–64. 25590022 10.5497/wjp.v2.i2.47 PMC4289909

[7] Li J, Li Z, Liang Z, Fabrication of a drug delivery system that enhances antifungal drug corneal penetration. Drug Delivery. 2018;25(1):938–949. 29658325 10.1080/10717544.2018.1461278 PMC6058611

[8] Subrizi A, del Amo EM, Korzhikov-Vlakh V, Tennikova T, Ruponen M, Urtti A. Design principles of ocular drug delivery systems: importance of drug payload, release rate, and material properties. Drug Discovery Today. 2019;24(8):1446–1457. 30738982 10.1016/j.drudis.2019.02.001

[9] Bertens CJF, Gijs M, van den Biggelaar FJHM, Nuijts RMMA. Topical drug delivery devices: a review. Exp Eye Res. 2018;168:149–160. 29352994 10.1016/j.exer.2018.01.010

[10] Rojanasakul Y, Robinson JR. Transport mechanisms of the cornea—characterization of barrier permselectivity. Int J Pharm. 1989;55(2–3):237–246.

[11] Cheng YH, Ko YC, Chang YF, Huang SH, Liu CJ. Thermosensitive chitosan-gelatin-based hydrogel containing curcumin-loaded nanoparticles and latanoprost as a dual-drug delivery system for glaucoma treatment. Exp Eye Res. 2019;179:179–187. 30471279 10.1016/j.exer.2018.11.017

[12] Zeng Y, Chen J, Li Y, Thermo-sensitive gel in glaucoma therapy for enhanced bioavailability: in vitro characterization, in vivo pharmacokinetics and pharmacodynamics study. Life Sci. 2018;212:80–86. 30268857 10.1016/j.lfs.2018.09.050

[13] Zhang Z, He Z, Liang R, Fabrication of a micellar supramolecular hydrogel for ocular drug delivery. Biomacromolecules. 2016;17(3):798–807. 26830342 10.1021/acs.biomac.5b01526

[14] Kim YC, Shin MD, Hackett SF, Gelling hypotonic polymer solution for extended topical drug delivery to the eye. Nat Biomed Eng. 2020;4(11):1053–1062. 32895514 10.1038/s41551-020-00606-8 PMC7655548

[15] Zhou Y, Fang A, Wang F, Core-shell lipid-polymer nanoparticles as a promising ocular drug delivery system to treat glaucoma. Chin Chem Lett. 2020;31(2):494–500.

[16] Weng Y, Liu J, Jin S, Guo W, Liang X, Hu Z. Nanotechnology-based strategies for treatment of ocular disease. Acta Pharm Sin B. 2017;7(3):281–291. 28540165 10.1016/j.apsb.2016.09.001 PMC5430571

[17] Occhiutto ML, Maranhão RC, Costa VP, Konstas AG. Nanotechnology for medical and surgical glaucoma therapya review. Adv Ther. 2020;37(1):155–199. 31823205 10.1007/s12325-019-01163-6 PMC6979457

[18] Li M, Lan J, Li X, Novel ultra-small micelles based on ginsenoside Rb1: a potential nanoplatform for ocular drug delivery. Drug Delivery. 2019;26(1):481–489. 30957571 10.1080/10717544.2019.1600077 PMC6461112

[19] Wang F, Bao X, Fang A, Nanoliposome-encapsulated brinzolamide-hydropropyl-beta-cyclodextrin inclusion complex: a potential therapeutic ocular drug-delivery system. Front Pharmacol. 2018;9:1–9. 29487529 10.3389/fphar.2018.00091 PMC5816959

[20] Zhai Z, Cheng Y, Hong J. Nanomedicines for the treatment of glaucoma: current status and future perspectives. Acta Biomater. 2021;125:41–56. 33601065 10.1016/j.actbio.2021.02.017

[21] Hu C, Sun J, Zhang Y, Local delivery and sustained-release of nitric oxide donor loaded in mesoporous silica particles for efficient treatment of primary open-angle glaucoma. Adv Healthcare Mater. 2018;7:1801047. 10.1002/adhm.201801047 30387326

[22] Kalomiraki M, Thermos K, Chaniotakis NA. Dendrimers as tunable vectors of drug delivery systems and biomedical and ocular applications. Int J Nanomed. 2016;11:1–12. 10.2147/IJN.S93069 PMC4694674 26730187

[23] Bravo-Osuna I, Noiray M, Briand E, Interfacial interaction between transmembrane ocular mucins and adhesive polymers and dendrimers analyzed by surface plasmon resonance. Pharm Res. 2012;29(8):2329–2340. 22565639 10.1007/s11095-012-0761-1 PMC3867740

[24] Bravo-Osuna I, Vicario-de-la-Torre M, Andrés-Guerrero V, Novel water-soluble mucoadhesive carbosilane dendrimers for ocular administration. Mol Pharmaceutics. 2016;13(9):2966–2976. 10.1021/acs.molpharmaceut.6b00182 27149661

[25] Kitchens KM, Kolhatkar RB, Swaan PW, Eddington ND, Ghandehari H. Transport of poly(amidoamine) dendrimers across Caco-2 cell monolayers: influence of size, charge and fluorescent labeling. Pharm Res. 2006;23(12):2818–2826. 17094034 10.1007/s11095-006-9122-2

[26] Esfand R, Tomalia DA. Poly(amidoamine) (PAMAM) dendrimers: from biomimicry to drug delivery and biomedical applications. Drug Discovery Today. 2001;6(8):427–436. 11301287 10.1016/s1359-6446(01)01757-3

[27] Nguyen DD, Luo L-J, Lai J-Y. Dendritic effects of injectable biodegradable thermogels on pharmacotherapy of inflammatory glaucoma-associated degradation of extracellular matrix. Adv Healthcare Mater. 2019;8(24):1900702. 10.1002/adhm.201900702 31746141

[28] Chauhan AS, Diwan PV, Jain NK, Tomalia DA. Unexpected in vivo anti-inflammatory activity observed for simple, surface functionalized poly(amidoamine) dendrimers. Biomacromolecules. 2009;10(5):1195–1202. 19348417 10.1021/bm9000298

[29] Mishra V, Jain NK. Acetazolamide encapsulated dendritic nano-architectures for effective glaucoma management in rabbits. Int J Pharm. 2014;461(1–2):380–390. 24291772 10.1016/j.ijpharm.2013.11.043

[30] Lancina MG, Singh S, Kompella UB, Husain S, Yang H. Fast dissolving dendrimer nanofiber mats as alternative to eye drops for more efficient antiglaucoma drug delivery. ACS Biomater Sci Eng. 2017;3(8):1861–1868. 29152562 10.1021/acsbiomaterials.7b00319 PMC5685521

[31] Lancina MG III, Wang J, Williamson GS, Yang H. DenTimol as a dendrimeric timolol analogue for glaucoma therapy: synthesis and preliminary efficacy and safety assessment. Mol Pharmaceutics. 2018;15(7):2883–2889. 10.1021/acs.molpharmaceut.8b00401 PMC6075655 29767982

[32] Kopeček J Hydrogel biomaterials: a smart future? Biomaterials. 2007;28(34):5185–5192. 17697712 10.1016/j.biomaterials.2007.07.044 PMC2212614

[33] Li Y, Rodrigues J, Tomás H. Injectable and biodegradable hydrogels: gelation, biodegradation and biomedical applications. Chem Soc Rev. 2012;41(6):2193–2221. 22116474 10.1039/c1cs15203c

[34] Holden CA, Tyagi P, Thakur A, Polyamidoamine dendrimer hydrogel for enhanced delivery of antiglaucoma drugs. Nanomed Nanotechnol Biol Med. 2012;8(5):776–783. 10.1016/j.nano.2011.08.018 21930109

[35] Desai PN, Yuan Q, Yang H. Synthesis and characterization of photocurable polyamidoamine dendrimer hydrogels as a versatile platform for tissue engineering and drug delivery. Biomacromolecules. 2010;11(3):666–673. 20108892 10.1021/bm901240g PMC2849659

[36] Kaga S, Arslan M, Sanyal R, Sanyal A. Dendrimers and dendrons as versatile building blocks for the fabrication of functional hydrogels. Molecules. 2016;21(4):497. 27092481 10.3390/molecules21040497 PMC6273238

[37] Buwalda SJ, Bethry A, Hunger S, Kandoussi S, Coudane J, Nottelet B. Ultrafast in situ forming poly(ethylene glycol)-poly (amido amine) hydrogels with tunable drug release properties via controllable degradation rates. Eur J Pharmaceut Biopharmaceut. 2019;139:232–239. 10.1016/j.ejpb.2019.04.006 30954658

[38] Wang J, He H, Cooper RC, Gui Q, Yang H. Drug-conjugated dendrimer hydrogel enables sustained drug release via a self-cleaving mechanism. Mol Pharmaceutics. 2019;16(5):1874–1880. 10.1021/acs.molpharmaceut.8b01207 PMC10958997 30974947

[39] Wang J, He H, Cooper RC, Yang H. In situ-forming polyamidoamine dendrimer hydrogels with tunable properties prepared via aza-Michael addition reaction. ACS Appl Mater Interfaces. 2017;9(12):10494–10503. 28263553 10.1021/acsami.7b00221 PMC5818279

[40] Lin C-C, Anseth KS. Glucagon-like peptide-1 functionalized PEG hydrogels promote survival and function of encapsulated pancreatic β-cells. Biomacromolecules. 2009;10(9):2460–2467. 19586041 10.1021/bm900420f PMC2745231

[41] Yang H, Tyagi P, Kadam RS, Holden CA, Kompella UB. Hybrid dendrimer hydrogel/PLGA nanoparticle platform sustains drug delivery for one week and antiglaucoma effects for four days following one-time topical administration. ACS Nano. 2012;6(9):7595–7606. 22876910 10.1021/nn301873v

[42] Xu L, Sheybani N, Yeudall WA, Yang H. The effect of photoinitiators on intracellular AKT signaling pathway in tissue engineering application. Biomater Sci. 2015;3(2):250–255. 25709809 10.1039/C4BM00245H PMC4335638

[43] Wang J, Williamson GS, Lancina MG III, Yang H. Mildly cross-linked dendrimer hydrogel prepared via aza-Michael addition reaction for topical brimonidine delivery. J Biomed Nanotechnol. 2017;13(9):1089–1096. 29479294 10.1166/jbn.2017.2436 PMC5819351

[44] Daly AC, Riley L, Segura T, Burdick JA. Hydrogel microparticles for biomedical applications. Nat Rev Mater. 2020;5(1):20–43. 34123409 10.1038/s41578-019-0148-6 PMC8191408

[45] Wang Z-K, Wang L-H, Sun J-T, Han LF, Hong CY. In situ generation of bioreducible and acid labile nanogels/microgels simply via adding water into the polymerization system. Polym Chem. 2013;4(5):1694–1699.

[46] Oh JK, Drumright R, Siegwart DJ, Matyjaszewski K. The development of microgels/nanogels for drug delivery applications. Prog Polym Sci. 2008;33(4):448–477.

[47] Thorne JB, Vine GJ, Snowden MJ. Microgel applications and commercial considerations. Colloid Polym Sci. 2011;289(5):625–646.

[48] Volpatti LR, Facklam AL, Cortinas AB, Microgel encapsulated nanoparticles for glucose-responsive insulin delivery. Biomaterials. 2021;267:120458. 33197650 10.1016/j.biomaterials.2020.120458

[49] Kim H, Shin M, Han S, Kwon W, Hahn SK. Hyaluronic acid derivatives for translational medicines. Biomacromolecules. 2019;20(8):2889–2903. 31251565 10.1021/acs.biomac.9b00564

[50] Abd El-Rehim HA, Swilem AE, Klingner A, Hegazy ESA, Hamed AA. Developing the potential ophthalmic applications of pilocarpine entrapped into polyvinylpyrrolidone–poly (acrylic acid) nanogel dispersions prepared by γ radiation. Biomacromolecules. 2013;14(3):688–698. 23414209 10.1021/bm301742m

[51] Laradji AM, Kolesnikov AV, Karakoçak BB, Kefalov VJ, Ravi N. Redox-responsive hyaluronic acid-based nanogels for the topical delivery of the visual chromophore to retinal photoreceptors. ACS Omega. 2021;6(9):6172–6184. 33718708 10.1021/acsomega.0c05535 PMC7948240

[52] Wang J, Cooper RC, He H, Li B, Yang H. Polyamidoamine dendrimer microgels: hierarchical arrangement of dendrimers into micrometer domains with expanded structural features for programmable drug delivery and release. Macromolecules. 2018;51(15):6111–6118. 30705466 10.1021/acs.macromol.8b01006 PMC6348485

[53] Wang J, Li B, Huang D, Nano-in-nano dendrimer gel particles for efficient topical delivery of antiglaucoma drugs into the eye. Chem Eng J. 2021;425:130498. 34121919 10.1016/j.cej.2021.130498 PMC8194049

